# Comparing Surgical Techniques for Meniscal Tears: A Systematic Review of Radiographic and Functional Outcomes

**DOI:** 10.7759/cureus.51239

**Published:** 2023-12-28

**Authors:** Haneen A Alhelali, Abdulrahman S Hassan, Faris A ALZahrani, Abrar A Aljubayri, Amjad A Aljubairy, Ahmed Alalasi, Ahmed S Alghamdi

**Affiliations:** 1 Orthopedic Surgery, King Fahad General Hospital, Jeddah, SAU; 2 Medicine and Surgery, University of Jeddah, Jeddah, SAU; 3 Medicine, Ibn Sina National College, Jeddah, SAU; 4 Orthopedic Surgery, King Abdullah Medical Complex, Jeddah, SAU; 5 College of Medicine, University of Jeddah, Jeddah, SAU

**Keywords:** arthritic changes, clinical scores, orthopedic surgery, outcomes, systematic review, surgical techniques, meniscal tears

## Abstract

Meniscal tears are a common orthopedic injury. The management approaches for meniscal tears include both surgical and non-surgical procedures; however, the majority of the surgeons opt for various surgical interventions. This systematic review aimed to compare the outcomes of different surgical techniques for meniscal tears. The systemic search was carried out in various databases including PubMed, Web of Science, CINAHL, and Scopus. Studies that investigated surgical techniques for meniscal repair and published between 2010 to 2023 were included. Out of the 7,421 potential studies identified from databases and Google Scholar search, only 17 studies were included in our systemic review. The follow-up periods ranged from 6 weeks to 123 months. Adverse effects were reported in some studies, including joint line tenderness, swelling, and loss of flexion, while others reported no significant adverse events. Pull-out repair and refixation techniques demonstrated better clinical outcomes and slower arthritic progression than partial meniscectomy. Mason-Allen stitches and simple stitches yielded comparable results, and both inside-out and all-inside techniques had similar clinical and functional outcomes. This systematic review provides valuable insights into the outcomes of different surgical techniques for meniscal tears. Further studies with longer follow-up periods may help assess the long-term effectiveness of these surgical techniques.

## Introduction and background

The menisci are vital structures within the knee joint that contribute to load distribution, joint stability, proprioception, and shock absorption [1]. Meniscal tissues are primarily composed of type I collagen fibers along with water. These components play a crucial role in the absorption of energy, as they facilitate the conversion of axial loading forces applied to the joint into hoop stresses inside the tissue [2]. With aging, the menisci degenerate as the cellularity and collagen content diminishes. This is why older individuals are more prone to meniscal tears. The overall prevalence of meniscal tears is estimated to be 60-70 per 100,000 individuals. However, younger patients with high functional demands such as athletes are also more likely to suffer from meniscal tears [3,4]. Meniscal tears are often associated with pain, meniscal extrusion, functional impairment, and long-term consequences if left untreated [5]. Osteoarthritis is also a deleterious consequence of meniscal injuries [6,7]. Meniscal tears often manifest as a result of acute trauma, degeneration, or a combination of both. However, it is important to recognize that not all meniscal tears are symptomatic. These asymptomatic tears usually heal spontaneously and do not need surgical intervention [8,9].

The management of meniscal tears has significantly evolved over the years. The choice of treatment approach for meniscal tears depends on several factors, including the age of the patient, physical activity level, tissue quality, and overall health status. Based on the severity of the tear, both non-operative and surgical approaches can be considered viable options [10]. It is important to carefully evaluate the individual circumstances and collaborate with a medical professional to determine the most suitable course of action [11]. Surgical interventions can be broadly categorized into meniscectomy and meniscus repair techniques [12]. Meniscectomy has historically been favored for the treatment of meniscal tears due to its surgical indications, which include rapid relief of symptoms, reduced length of hospital stay, and improved quality of life. However, it is important to consider that meniscectomy is also associated with significant long-term degenerative changes in the knee joint. Therefore, the decision to proceed with meniscectomy should be based on careful consideration of the patient's individual circumstances, including the severity of the meniscal tear, the patient's age, activity level, and the presence of other knee pathologies [13]. According to the European Society of Sports Traumatology, Knee Surgery, Arthroscopy (ESSKA) consensus report, arthroscopic partial meniscectomy should not be considered as the first line of treatment [14]. Meniscus repair techniques, on the other hand, aim to preserve the meniscal tissue and reduce the adverse consequences of meniscectomy [12]. Previously, various studies have assessed the comparative effects of different surgical approaches; however, the heterogeneity in surgical approaches, patient characteristics, and study methodologies has made it challenging to draw clear comparisons among these techniques.

## Review

Methods

For this systematic review, we followed the Preferred Reporting Items for Systematic Reviews and Meta-Analyses (PRISMA) guidelines [15].

Search Strategy and Data Sources

A comprehensive search strategy was developed to identify relevant studies on surgical procedures for meniscal tears. We searched multiple databases, including CINAHL Plus, PubMed, Scopus, and Web of Science. The search terms used a combination of keywords such as "meniscal," "tears," "wound," "laceration," "wound healing," and "repair." The detailed search strategy with specific combinations of keywords is provided in Appendix A. In addition to the databases, we also searched Google Scholar to expand the body of evidence. The inclusion criteria for the studies were as follows: (i) they investigated surgical treatment for meniscal tears, (ii) they were published in or after 2010, and (iii) they were published in English.

Study Selection and Risk-of-Bias Assessment

After retrieving the search results and removing duplicate studies, the remaining studies were imported into the reference manager, EndNote, for further screening. Two independent reviewers conducted the study selection process in a blinded manner using the titles and abstracts of the articles. Studies that did not meet the inclusion criteria were excluded at this stage. Any discrepancies between the two reviewers were resolved through discussion, and a third reviewer was consulted if necessary.

To assess the risk of bias in the included studies, we will use appropriate tools such as the Cochrane Risk of Bias tool or the Newcastle-Ottawa Scale, depending on the study designs.

Data Extraction and Analysis

Data from the included studies will be extracted using a standardized form created by the authors. The extracted data will include demographic information, details of the surgical procedures adopted, outcome measures, and effect measures of the interventions. We will also extract data related to the risk-of-bias assessment. If necessary, we will contact the authors of the included studies to obtain additional information or clarification.

Due to the anticipated heterogeneity in study designs and outcome measures, a narrative synthesis of the findings is planned. If the included studies are sufficiently similar in terms of population, interventions, and outcome measures, a meta-analysis will be conducted using appropriate statistical methods. After removing the duplicate studies, the final file was exported to Rayyan (systemic review screening software) [16].

Results

Included Studies

A total of 7,421 potential studies were identified during the database search. The number of studies identified from each database were as follows: PubMed (n=1912), CINAHL Plus (n=962), Web of Science (n=2010), and Scopus (n=2468). The search in Google Scholar revealed 69 studies. Following the removal of duplicate studies, a total of 4,679 records were further assessed. After assessing the titles and abstracts of the articles, 3,698 were further excluded. From the remainder studies, only 17 studies were finally eligible for inclusion in the systemic review.

Flow Diagram

**Figure 1:**
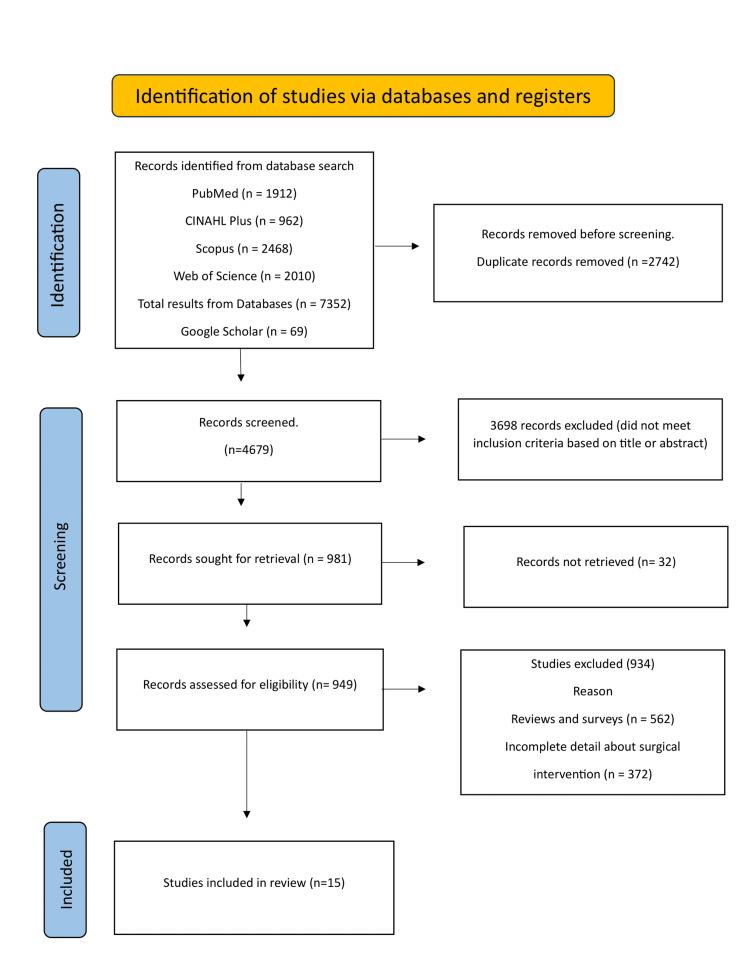
PRISMA study selection flow chart PRISMA, Preferred Reporting Items for Systematic Reviews and Meta-Analyses

Figure 1 shows the PRISMA flow diagram of the systemic review. The figure explains the reasons for the exclusion of non-relevant studies.

A total of 17 studies published between 2010 to 2023 were included in the final analysis of this systemic review. A total of 1,650 participants were included in all the studies. Majority of the studies included in the study had a small sample size (below 100 participants), with only five studies having participants above hundred [17-21]. The surgical techniques examined in these studies included pull-out repair, Fast-Fix, Mason-Allen stitch, partial meniscectomy, and more. The outcome measures used in these studies included various clinical scoring systems such as the International Knee Documentation Committee (IKDC), Tegner Lysholm Knee Score, Western Ontario Meniscal Evaluation Tool (WOMET), Knee injury and Osteoarthritis Outcome Score (KOOS), and Visual Analog Scale (VAS) for pain assessment. The follow-up periods ranged from 6 weeks to 123 months. Adverse effects were reported in some studies, including joint line tenderness, swelling, and loss of flexion, while others reported no significant adverse events. A detailed summary of findings of the included studies is mentioned in Table 1.

**Table 1:** Descriptive details of all the studies included in this systemic review ACL, anterior cruciate ligament; CI, confidence interval; IKDC, International Knee Documentation Committee; MMPRTs, medial meniscus posterior root tear; MMRT, medial meniscus root tear; MRI, magnetic resonance imaging; SF-12, 12 Item Short Form Health Survey; TKR, total knee replacement; VAS, visual analog scale; WOMAC, McMaster Universities Osteoarthritis Index; WOMET, Western Ontario Meniscal Evaluation Tool M, R, and S groups refer to the meniscectomy, repair, and sham surgery groups, respectively.

Author	Year	Study design	No. of participants	Suture technique/surgical treatment	Outcome measure	Post-operative rehabilitation	Follow-up	Adverse effects	Main findings	Conclusion
Ahn et al. [22]	2010	Retrospective, comparative, cross-sectional, clinical study	38	Pull-out repair	IKDC and Tegner Lysholm Knee Score	_	24 months	Adverse effects were not reported in the study.	The MMRT pull-out repair group demonstrated statistically significant improvements in both the Tegner and Lysholm activity scale and IKDC score (p = 0.017 and p< 0.001, respectively). No statistically significant differences were seen between the alignment and conservative groups (p=0.487).	The intervention group demonstrated better outcomes compared to conservative approaches.
Tachibana et al. [23]	2010	Retrospective study	46	Fast-Fix technique	Second look arthroscopy	For two weeks, the knee was immobilized at 0 degrees of extension in a hinged long leg brace. As soon as the knee could withstand it, partial weight-bearing with crutch support was permitted. Exercises were allowed after 2 weeks, and at 3 weeks, full weight-bearing with discarded crutches was allowed. Spots were not allowed for 8 to 10 months, while pivoting and squatting were not allowed for 3 months.	3 months	Joint tenderness was reported in seven patients, whereas persistent swelling was seen in one patient in knee area. Three patients had a loss of flexion at ≥10^o^.	48% (30) of menisci demonstrated full healing of the originally formed tear sites without forming new tears.	Fast-fix meniscal repair in conjunction with the reconstruction of the anterior cruciate ligament was successful in almost 74% of the cases. Approximately 83% of the meniscal repair was symptom-free regardless of the integrity of the meniscal.
Kim et al. [24]	2011	A comparative, prospective study	58 (M group, n=28), (R group, n=30)	Partial meniscectomy and pull-out repair	IKDC and Tegner Lysholm Knee Score	_	24-65 months	Two patients lost the fixation strength, while two patients lost restoration of hoop tension	Both groups showed improved levels of IKDC and Lysholm scores (p=0.5). However, the Repair group had better IDKC and Lysholm scores, as well as lower joint progression and narrowing of the Kellgren-Lawrance grade compared to group M	An MRI and second-look arthroscopy revealed sound healing with restoration of the meniscus's hoop tension, and arthroscopic pull-out repair of a medial meniscal reconstruction produced noticeably superior clinical and radiologic outcomes than partial meniscectomy. The findings suggested that the arthroscopic pull-out repair technique is a useful therapy for MMRT.
Sihvonen et al. [17]	2013	Sham-controlled, randomized, multicenter, and double-blind trial	146	Sham surgery and arthroscopic partial meniscectomy	Tegner Lysholm Knee Score and WOMET	_	12 months	Adverse events were seen in one patient who underwent partial meniscectomy. Deep infection of the index knee was observed after 4 months.	The Lysholm score was 21.7 in the partial meniscectomy group as compared to 23.3 points in group S (95% CI: -7.2 to 4.0). The WOMET scores were 27.1 and 24.6, respectively (95% CI: -9.2 to 4.1), and the knee pain scores after physical exercise were 3.3 and 3.1 points, respectively (95% CI: -0.9 to 0.7)	The results of a partial meniscectomy performed via arthroscopy were identical and superior to those of a sham procedure.
Yim et al. [18]	2013	Randomized controlled trial, level I evidence	102	Arthroscopic partial meniscectomy and physical therapy	Lysholm	Partial weight-bearing exercises were carried out for almost 6 weeks. Closed kinetic chain strengthening exercises and full weight-bearing exercises were permitted after 6 weeks of surgery. Light running was permitted after 3 and sports after 6 months. Progressive and strengthening exercises were permitted within the tolerable range.	6 weeks to 123 months	Adverse effects were not mentioned in the study	At the final follow-up (2 years), Lysholm knee scores were 84.3 and 83.2 in the non-operative management and meniscectomy groups, respectively (p = 0.237).	There were no statistically significant differences observed between non-operative management and arthroscopic meniscectomy with stretching and strengthening exercises in terms of satisfaction, relief of knee pain, and knee function in a 2-year follow-up.
Katz et al. [20]	2013	Randomized controlled trial	351	Arthroscopic partial meniscectomy and physical therapy	WOMAC	-	6-12 months	Serious adverse events were reported in three patients in the surgery group and in two patients in the physical therapy arm	At 6 months, WOMAC score was 20.9 in the surgical group and 18.5 in the non-surgical group	No significant difference was seen in both groups
Lee et al. [25]	2014	A retrospective comparative study, level III evidence	50 (n=25 Mason-Allen group; M), (n=25, Controlled group; S)	Mason –Allen stich and simple stich	IKDC and Tegner Lysholm Knee Score	Knee exercises within a range of motion by the use of a continuous passive motion machine, and isometric exercises after a day of surgery. Partial weight-bearing with the aid of a crutch was allowed for 6 weeks.	3 to 36 months	Adverse effects were not mentioned in the study	The repaired meniscal tear tended to heal better in the M group than in the S group (p=0.065). Postoperative clinical outcomes did not differ between the two groups.	Mason–Allen stitches have an improved degree of meniscal extrusion when compared with simple stitches.
Chung et al. [26]	2015	Retrospective, comparative study, level III evidence	57 (M, n=20), (R, n=37)	Partial meniscectomy and pull-out repair technique		_	5 years	Adverse effects were not mentioned in the accessed article.	Results of this study reported that the R group had significantly better IDKC and Lysholm scores (p = 0.002 and p < 0.002, respectively) than the M group	Refixation was found to be more effective for MMPRTs than partial meniscectomy in terms of radiological and clinical survival outcomes for a 5-year follow-up. Refixation did not prevent the progression of arthrosis completely but slowed the progression of arthritic changes.
Pan et al. [27]	2015	Prospective, comparative study	31	Pull-out repair and conservative	IKDC and Tegner Lysholm Knee Score	_	3-26 months	Adverse effects were not reported in the study.	The difference between IDKC and Lysholm scores was not statistically significant for the two groups. However, after operative treatment, patients had higher functional scores and lower osteoarthritis with a significance of p< 0.05.	Both techniques have effectively improved the knee function but surgical technique has improved functional scores of the knee and lowered the osteoarthritis.
Tjoumakaris et al. [28]	2015	Prospective evaluative study	9	Pull-out and repair techniques	WOMAC and Lysholm	_	30 months	Adverse impacts were not reported	Extrusion averaged 1.0 mm in patients with evidence. The Lysholm and WOMAC scores were 81.6 and 11.2, respectively. No correlation was found and series scores.	Four patients showed a recurrence of tears. There was also an increase in the peripheral meniscus tear far from the repairing site, indicating the excessive stress induced by the repair.
LaPrade et al. [29]	2017	Cohort study, level III evidence	50 (15 lateral, 35 medial)	Lateral versus medial pull-out repair	Tegner Lysholm Knee Score, WOMAC, SF-12	Non-weight-bearing exercises were allowed for the first 6 weeks. Quadriceps strengthening and passive knee range-of-motion exercises were allowed after a day of surgery. Partial weight-bearing exercises were allowed after 7 weeks. Strength and endurance exercises were allowed after 2 months. Patients can come back to normal routine after 6 months.	2 years	Not reported	All failures occurred in patients <50 years of age and those who underwent medial root repair. There was no significant difference in failure based on laterality and age (p = 0.541 and p = 0.544, respectively).	After surgery, posterior meniscal root outcomes were improved significantly. The transtibial double-tunnel pull-out meniscal repair improved patient satisfaction.
Chung et al. [30]	2017	Case-control study, level III evidence	39 (23 increased extrusion, 16 decreased extrusion)	Meniscus repair, increased versus decreased extrusion	IKDC and Tegner Lysholm Knee Score	_	5 years	Not reported	The results of this study demonstrate a substantial rise in meniscus extrusion in group A (repair group), with the mean (±SD) increasing from 3.5 ± 0.9 mm before surgery to 5.1 ± 1.4 mm at 1 year postoperatively (p < 0.001). Conversely, in group B (meniscectomy group), there was a significant drop in meniscus extrusion from 4.1 ± 1.3 mm before surgery to 3.5 ± 1.4 mm at 1 year after surgery (p < 0.001).	The results of this study demonstrate that in MMPRT patients, pull-out fixation leads to satisfactory midterm outcomes regardless of the extrusion method at 1-year follow-up.
Furumatsu et al. [31]	2019	A comparative study	39	FasT-Fix versus FasT-Fix modified Mason-Allen stitch	Lysholm, VAS, and KOOS	_	1 year	No reported	KOOS and VAS pain scores and arthroscopic meniscal healing scores of F-MMA pull-out repair were superior to single Fast-Fix pull-out repairs.	F-MMA suture configuration has obtained better meniscal healing and improved clinical outcomes when compared with the single Fast-Fix pull-out repair in people with MMPRTs.
Abdel Tawab Abdallah et al. [8]	2020	Hospital-based prospective study	61	Inside-out technique, all inside and outside-in techniques	IKDC and Tegner Lysholm Knee Score	Partial weight-bearing through crutches, active knee extension and flexion for 6 weeks with a range of motion, stretching, and strengthening exercises for calf muscles and quadriceps with full extension ACL braces for 6 weeks, and passive knee extension and flexion with a motion range of 0-90 gradually for 6 weeks. Quadriceps and hamstring strengthening exercises were performed at 6 weeks	6-12 months	Three patients had a failure to repair	On radiological evaluation, 11 patients show non-healed repair despite having no other clinical symptoms. Three cases presented with MRI grade III intensity, showed clinical symptoms, and underwent revision partial meniscectomy.	The clinical and radiological outcomes of the patients who had MRI follow-ups for 6 months (30 knees) were correlated in all surgical techniques.
Katz et al. [21]	2020	Longitudinal study	351	Arthroscopic partial meniscectomy, physiotherapy	KOOS, TKR	-	5 years	Not reported	The hazard ratio was 2.0 (95% CI: 0.8, 4.9) in arthroscopic partial meniscectomy for total knee replacement compared to physiotherapy in the intent-to-treat group.	TKR is more common in arthroscopic partial meniscectomy compared to the non-operated group.
Rathava et al. [12]	2021	Prospective study	30	Inside-out technique, all inside and outside-in techniques, the hybrid technique was performed arthroscopically	IKDC and Tegner Lysholm Knee Score	Full weight-bearing from the day onward of the surgery, active range of motion from the second day of motion, and strengthening exercises for quadriceps after recovery from anesthesia.	6-12 months	Hemarthrosis was seen in one case, and superficial stitch infection was seen in one case.	The pull-out technique seems to be superior as compared to other techniques as it offers a high rate of meniscus healing without extended time for the operation.	All the repair techniques used for meniscus tears yielded comparative functional and clinical outcomes, and the results of the techniques are not statistically significant. There were good to excellent results in 99.66% of the cases.
Borque et al. [19]	2023	Cohort study, level III evidence	192	All-inside versus Inside-out	_	_	2 years	Not reported	In the first year, 8% of lateral meniscal tears and 16% of medial meniscal tear repairs failed with the inside-out technique and 42% with all-inside techniques.	All inside repair led to a higher rate of failure than inside-out repair of meniscal tears in elite athletes. A higher failure rate was observed in medial than in lateral meniscal repair.

Discussion

The aim of this systematic review was to evaluate the efficacy of different surgical approaches in improving patient outcomes. To address the objective of comparing outcomes, we will begin by summarizing the key findings related to meniscal repair and meniscectomy. Several studies included in this review, such as Ahn et al. [22], Kim et al. [24], and Chung et al. [26], suggest that pull-out repair is an effective technique for treating meniscal tears compared to partial meniscectomy. These studies reported improvements in clinical outcomes, as indicated by higher IKDC and Lysholm scores, in patients undergoing pull-out repair compared to conservative or partial meniscectomy approaches. These findings are consistent with a systemic review and meta-analysis by Ro et al. [32], who also concluded that meniscal repair has higher efficacy compared to partial meniscectomy for medial meniscal tears.

Additionally, Tachibana et al. [23] examined the Fast-Fix technique, a form of meniscal sutures along with anterior cruciate ligament reconstruction, and reported successful outcomes in two-thirds of the patients, with the majority experiencing symptom relief. The Fast-Fix technique offers advantages as it does not require a secondary safety incision, reducing the morbidity associated with the procedure [33]. Furthermore, studies by Lee et al. [25], Tjoumakaris et al. [28], and Furumatsu et al. [31] investigated the impact of different suture techniques on outcomes. Although these techniques may affect meniscal extrusion and healing, no consistent significant differences were found in terms of clinical outcomes and patient satisfaction.

On the other hand, the comparison of meniscal repair and meniscectomy with non-surgical techniques is also relevant to our research question. Previous findings have indicated that partial meniscectomy is not superior to non-surgical techniques [34]. Similarly, a meta-analysis of six randomized controlled trials found no significant difference between partial meniscectomy combined with physical therapy and physical therapy alone [35]. However, they concluded that partial meniscectomy combined with physical therapy can help reduce pain and improve patient outcomes [36]. Conversely, Ma et al. [37], in their meta-analysis, revealed that partial meniscectomy combined with physical therapy showed significantly better pain control for six months compared to physical therapy alone.

Several comparative studies, including Yim et al. [18], Lee et al. [25], and Pan et al. [27], compared different surgical techniques with conservative management or partial meniscectomy. These studies generally found that while surgical techniques yielded some improvements in knee function and pain relief, the differences were not statistically significant. Furthermore, the location of the meniscal tear may influence the choice of surgical technique. For example, LaPrade et al. [29] examined the outcomes of lateral versus medial pull-out repair and found significantly improved outcomes for posterior meniscal root tears. Chung et al. [30] and Abdel Tawab Abdallah et al. [8] investigated the impact of meniscus extrusion on outcomes and found that meniscus extrusion had a significant effect on results. Therefore, it is crucial to consider these factors during surgical planning.

Regarding adverse effects and complications associated with the surgical techniques, most studies did not report significant issues. However, some cases of non-healed repairs, recurrent tears, and other complications were mentioned. Additionally, only six studies included in this systematic review provided details of rehabilitation protocols, which included various weight-bearing exercises, light running, and a gradual progression toward strength exercises. A systematic review by Harput et al. reported that 78% of studies agreed that athletes can return to sport between three to six months [38].

Limitations

This study on surgical approaches for meniscal tears has several limitations. Firstly, the absence of an evaluation of the risk of bias in the included studies raises concerns about the overall reliability of the findings. The search strategy is deemed excessively sensitive, potentially introducing irrelevant studies into the analysis. Additionally, the eligibility criteria lack clarity, and there is a lack of documentation regarding the preliminary analysis of identified studies. With only 17 studies included from an initial pool of 7,421, questions arise about the representativeness of the selected studies. Heterogeneity in surgical techniques and outcome measures, incomplete reporting of adverse effects, and limited focus on rehabilitation details further diminish the study's robustness. Varied follow-up periods and a lack of consideration for publication bias are additional limitations. Acknowledging and addressing these limitations is crucial for a nuanced interpretation of the study's findings and to guide future research in the field.

## Conclusions

In summary, this systematic review provides a comprehensive analysis of the existing literature on surgical approaches for meniscal tears, encompassing a total of 17 eligible studies. The synthesis of evidence suggests that pull-out repair emerges as a favorable technique, demonstrating superior outcomes compared to partial meniscectomy in various clinical scoring systems, such as IKDC and Lysholm scores. The Fast-Fix technique and different suture techniques also show promise, with notable success rates and patient symptom improvement. Notably, the findings challenge the traditional belief in the superiority of partial meniscectomy, revealing comparable outcomes between non-operative techniques and arthroscopic partial meniscectomy. This aligns with the growing body of evidence advocating for non-surgical approaches, particularly in degenerative meniscal tears, emphasizing the success of physical therapy in managing asymptomatic cases. The impact of meniscal tear location, whether lateral or medial, and the presence of meniscus extrusion emerge as crucial considerations influencing surgical decisions and outcomes. Additionally, the review underscores the importance of a thorough understanding of each surgical technique's nuances, considering factors such as meniscal laterality, extrusion, and tear location. While most studies did not report significant adverse effects, it is essential to acknowledge the potential for non-healed repairs, recurrent tears, and other complications. The limited reporting on rehabilitation details further highlights the need for standardized guidelines to facilitate optimal postoperative care and patient recovery. In light of these findings, orthopedic surgeons are encouraged to consider the nuanced variations in outcomes based on surgical techniques, extrusion, and meniscal laterality when planning interventions. This systematic review provides valuable insights into the ongoing discourse on the management of meniscal tears, emphasizing the importance of evidence-based decision-making in enhancing patient outcomes and guiding future research in this field.

## Appendices

Appendix A

**Table 2:** Terms and strategy used for literature search

Terms and strategy
#1 meniscal [TI/AB]
#2 tears [TI/AB]
#3 wound [TI/AB]
#4 laceration [TI/AB]
#5 injury [TI/AB]
#6 tear [TI/AB]
#7 OR/2-6
#8 surgical techniques [TI/AB]
#9 wound healing [TI/AB]
#10 repair [TI/AB]
#11 inside-out suture technique [TI/AB]
#12 all-inside with absorbable sutures [TI/AB]
#13 OR/8-12
#14 1 AND 7 AND 13
